# Factors for Lower Walking Speed in Persons with Multiple Sclerosis

**DOI:** 10.1155/2013/875648

**Published:** 2013-03-31

**Authors:** Leandro Alberto Calazans Nogueira, Luciano Teixeira dos Santos, Pollyane Galinari Sabino, Regina Maria Papais Alvarenga, Luiz Claudio Santos Thuler

**Affiliations:** ^1^Neurology Postgraduate Program of Federal University of Rio de Janeiro State, Rua Mariz e Barros 775, 20270-004 Rio de Janeiro, Brazil; ^2^Federal Institute of Education, Science and Technology of Rio de Janeiro, Rua Professor Carlos Wenceslau, 343, 21715-000 Rio de Janeiro, Brazil; ^3^Departamento de Neurologia, Physical Therapy of Gaffrée e Guinle University Hospital, Rua Mariz e Barros 775, 20270-004 Rio de Janeiro, Brazil; ^4^National Cancer Institute (INCA), Praça Cruz Vermelha, 23, 20230-130 Rio de Janeiro, Brazil

## Abstract

*Objective*. The purpose of this study was to analyze factors related to lower walking speed in persons with multiple sclerosis (PwMS). *Methods*. A cross-sectional survey was conducted. The study participants were 120 consecutive PwMS, who were able to walk, even with device assistance. Demographic and clinical data were collected. Walking speed was measured in 10 m walk test. Possible factors were assessed: disability, fatigue, visual functioning, balance confidence, physical activity level, walking impact, cognitive interference, and motor planning. A forward linear multiple regression analysis examined the correlation with lower speed. *Results*. Lower walking speed was observed in 85% of the patients. Fatigue (41%), recurrent falls (30%), and balance problems were also present, even with mild disability (average EDSS = 2.68). A good level of physical activity was noted in most of the subjects. Dual-task procedure revealed 11.58% of walking speed reduction. Many participants (69.57%) imagined greater walking speed than motor execution (mean ≥ 28.42%). Physical activity level was the only characteristic that demonstrated no significant difference between the groups (lower versus normal walking speed). Many mobility measures were correlated with walking speed; however, disability, balance confidence, and motor planning were the most significant. *Conclusions*. Disability, balance confidence, and motor planning were correlated with lower walking speed.

## 1. Introduction

Persons with multiple sclerosis (PwMS) present difficulties in mobility, self-care, and domestic life [1]. Many researchers have found lower walking speed in the PwMS group, when compared with the control group [2–4]. Difficulty in walking is the most visible sign of functional impairments caused by multiple sclerosis (MS) [5]. Among PwMS, 41% reported having difficulty in walking, and 13% related inability to walk at least twice a week. Of those with difficulty in walking, 70% stated that it was the most challenging aspect of having MS [6]. It has been shown that gait and balance impairment may begin to deteriorate in the early stages of the disease, even in the absence of clinical signs of pyramidal dysfunction [7]. 

Walking is a complex functional activity; thus, many variables contribute to or influence walking speed. Fritz and Lusardi [8] reported that these include an individual's health status, motor control, muscle performance and musculoskeletal condition, sensory and perceptual function, endurance and habitual activity level, cognitive status, and motivation and mental health, as well as the characteristics of the environment in which one walks. Some of those factors have been shown to negatively influence the walking speed of PwMS: muscle performance [9], sensorial function [10], habitual activity level [11], and disability [4]. Disability-related factors of PwMS were the primary indicators of use of physical therapy services [12]. 

Balance impairment [13] and recurrent falls [14] are frequent findings in PwMS, because visual dysfunction is one of the most common clinical manifestations of MS [15]. PwMS have both motor and cognitive impairment, making them vulnerable when performing dual tasks. Motor imagery (MI) is widely used to study cognitive aspects of the neural control of action [16], similar to motor planning in the absence of sensory feedback [17]. 

Typically, the reduction in walking speed represents a cautious strategy on the performance of such a dynamic task and it is affected by several factors. Walking speed was found to show the strongest correlations with other mobility measures in PwMS [18]. Many approaches have been used to improve walking, and positive results of interventions have been described; however, there is a lack of studies that combine multiple factors with walking performance. The purpose of the present study was to analyze factors related to lower walking speed in PwMS.

## 2. Methods

### 2.1. Patients

A cross-sectional study was conducted at Lagoa Hospital, Rio de Janeiro, Brazil, from June to October 2011. Lagoa Hospital is an outpatient referral center for PwMS. Patients with confirmed diagnosis of MS, according to the criteria established by McDonald et al. [19], who were independently walking or walking with assistance (i.e., cane, crutch, or walker) were invited to participate in the study. The exclusion criteria were requirement of wheelchair for mobility and a relapse history during the previous 30 days. A total of 120 consecutive PwMS were included. An institutional review board approved the procedure, and all participants provided written informed consent documentation. 

### 2.2. Procedure

The participants completed a demographic questionnaire, including a history of falls, and patients with recurrent falls (more than one fall in previous year) were classified as fallers. Subsequently, the participants were submitted to clinical neurological assessment for disability classification using the Expanded Disability Status Scale (EDSS), and walking assessment by clinical testing. They also filled self-applicable questionnaires for possible factors on walking impairment, such as fatigue (Modified Fatigue Impact Scale), vision-Specific quality of life (National Eye Institute Vision Functioning Questionnaire-25), balance confidence (The Activities-specific Balance Confidence Scale), level of physical activity (International Physical Activity Questionnaire), and walking impact (Multiple Sclerosis Walking Scale-12). 

### 2.3. Main Outcome Measures

#### 2.3.1. Gait Questionnaire

The Multiple Sclerosis Walking Scale-12 (MSWS-12) is a 12-item self-report measurement of the impact of MS on walking. The items are rated on a 5-point scale from 1 (*Not at all*) to 5 (*Extremely*) and represent limitations in walking during the past 2 weeks. The MSWS-12 is scored by summing the item scores, subtracting 12 from the sum, dividing the difference by 60, and then multiplying the result by 100. The scores range between 0 and 80, and higher scores indicate worse walking mobility or more walking difficulty [20]. The MSWS-12 has good evidence for internal consistency, test-retest reliability, and validity of scores as a measure of walking mobility in MS [21].

#### 2.3.2. Gait Clinical Trial

The participants were instructed to walk barefoot at their self-selected, comfortable speed along a 14 m walkway. A ‘‘dynamic start” was used where the subject might accelerate 2 m before entering the timed 10 m distance and decelerate 2 m afterward. As long as subjects are able to ambulate the required 14 m, they are able to participate in the test. Timing was started when the lead foot crossed the starting line and was stopped when the lead foot crossed the finish line. Speed was only calculated for the 10-m distance between the starting line and finish line to avoid measuring the acceleration and deceleration phases of gait. The second walking trial was recorded to minimize the learning effect. The walking time was registered and then the gait speed was estimated. The 10 m timed walk test (10 m-TWT) is valid and reliable for patients with neurologic impairment [22]. Paltamaa et al. [23] described a good test-retest and interrater reliability in PwMS, which have been used in longitudinal [24] and survey [1] studies.

*Physical Activity.* Physical activity was measured using the short form of the International Physical Activity Questionnaire (IPAQ), which was designed for population surveillance of physical activity among adults. The IPAQ short form has six items that measure the frequency and duration of vigorous-intensity activities, moderate-intensity activities, and walking during a 7-day period. The respective frequency values for vigorous, moderate, and walking activities were multiplied by 8, 4, and 3.3 metabolic equivalents and then summed to form a continuous measure of physical activity [25]. Weikert et al. [26] found a strong correlation between IPAQ scores and accelerometer movement counts in PwMS. The IPAQ was validated for use in the Portuguese language [27]. 

*Fatigue.* Fatigue was assessed by the Modified Fatigue Impact Scale (MFIS), which is a 21-item self-report multidimensional scale developed to assess the perceived impact of fatigue on a variety of daily activities over the previous 4 weeks. The MFIS total score is the sum of the three subscales, ranging from 0 to 84. All items are scaled so that higher scores indicate a greater impact of fatigue on a patient's activities [28]. Values ≤38 indicate the absence of fatigue [29]. The reliability and validity of the MFIS have been established in PwMS, and the MFIS has been validated for use in the Portuguese language [30].

#### 2.3.3. Perceived Balance Confidence

Perceived Balance Confidence was assessed using the Activities-Specific Balance Confidence (ABC) scale. This 16-item scale requires respondents to self-rate their balance confidence in performing activities of daily living from 0 to 100%. The ratings are averaged to derive the total scores, and higher scores reflect higher levels of balance confidence [31]. The ABC scale has been used with various populations and its use in PwMS [32] has been supported by psychometric evidence. 

#### 2.3.4. Dual Task

The subjects were instructed to perform 10m TWT while executing an arithmetic task, namely, counting aloud backward from 100, subtracting by 3, to manipulate the attention demands of the subjects during a motor task. One investigator walked beside the PwMS adjacent to the walkway to provide support if a loss of balance occurred. Gait speed and cadence were measured as 10m TWT. 

#### 2.3.5. Motor Planning

The motor planning was measured bymental chronometry. This strategy is based on the observation that the duration of mentally simulated and executed motor tasks is comparable. Thus, by knowing the time length of the physical act, the investigator asked the patient to signal the beginning and termination of the imagery performance. A comparable time period of the imagery and physical performance of the task was considered to be evidence of engagement in motor imagery practice of the required task. The subjects were instructed to imagine themselves (first-person perspective) walking through the walkway, and, subsequently, kinesthetic motor imagery was used [33]. Bakker et al. [16] showed that kinesthetic motor imagery has higher correspondence with gait execution than visual motor imagery. The motor planning results were obtained from the ratio between walking imagination time and walking execution time. 

#### 2.3.6. Vision-Specific Quality of Life

The 25-item National Eye Institute Visual Function Questionnaire (NEI VFQ-25) was self-administered in all participants to assess self-reported, vision-specific quality of life. The NEI VFQ-25 Brazilian version showed reliable and valid psychometric properties [34]. The NEI VFQ-25 consists of 12 vision-targeted subscales: general health, general vision, ocular pain, near activities, distance activities, social functioning, mental health, role difficulties, dependency, driving, color vision, and peripheral vision. Each subscale is converted to a score from 0 (*lowest*) to 100 (*highest*) [35]. The NEI VFQ-25 was administered and calculated according to standard instructions; patients were requested to answer all questions as though they were wearing their usual correction (glasses or contact lenses) for the visual activity specified. Average and standard deviation were calculated for each subscale. 

### 2.4. Statistical Analysis

Normal probability plots were inspected for each variable. Data distribution of each variable was verified through the Shapiro-Wilk test. The sample was dichotomized according to walking speed. The walking speed cut-off used was the recent normative data stratified by age and gender described by Bohannon and Williams Andrews [36]. The comparison between the groups was performed using the nonpaired Student's *t*-test or the Mann-Whitney *U* test, according to the data distribution. The Chi-square test was used to analyze categorical variables. Pearson's and Spearman's rank correlations were used between walking speed and possible factors for lower speed, when appropriate. A correlation above 0.90 was interpreted as very high, 0.70 to 0.89 as high, 0.50 to 0.69 as moderate, 0.30 to 0.49 as low, and less than 0.29 as little, if any, correlation [37]. A forward linear multiple regression analysis was performed for each of the significant variables from the correlation and walking speed entered as independent and dependent variables, respectively, described by the percentage of normative strata. Significance level was established at 5%  (*P* < .05). All data were analyzed using the Statistical Package for Social Sciences (SPSS, Inc., Chicago, IL, USA) Version 17.0 software package, and graphic analyses were performed using GraphPad Prism (GraphPad Software, San Diego, CA, USA) Version 5.00 for Windows. 

## 3. Results

### 3.1. Sample Characteristics

The majority of the participants were young adults with a mean age of 38.14 years (SD ±12.32), and most of them were female (74.17%) with normal body fat (mean body mass index = 23.11). Relapsing-remitting was the most frequent evolution form observed among 82.50% of the participants, followed by the secondary progressive (10.83%) and primary progressive (6.67%) forms. Mild disability was observed in most of the PwMS with an EDSS mean of 2.68 (SD ±2.00). 

### 3.2. Descriptive Statistics

Lower walking speed was observed in 85.00% of the subjects, recurrent falls in 30.00%, self-report fatigue in 40.83%, and balance confidence in 72.13% (SD ±26.17), with good level of physical activity observed in most of the samples (mild intensity—35.83%; moderate intensity—25.83%; vigorous intensity—38.33%). Dual-task procedure revealed 11.58% of walking speed reduction, whereas PwMS with lower walking speed values comprised 9.30% and those with normal walking speed were 15.71%. Many participants (69.57%) imagined greater walking speed than motor execution (mean ≥28.42%). The participants were dichotomized according to walking speed. Physical activity level was the only characteristic that demonstrated no significant difference between the groups (lower versus normal walking speed). The descriptive statistics are provided in Table 1.

### 3.3. Bivariate Correlation Analysis

Table 2 presents the main correlations among walking speed and EDSS (*r* = −0.740), balance confidence (*r* = 0.703), self-perceived walking impact (*r* = −0.677), motor planning (*r* = 0.556), recurrent falls history (*r* = 0.445), perceived fatigue (*r* = −0.423), and physical activity level (*r* = 0.315). All correlations were found to be significant (*P* < .01). Vision-specific quality of life subscales showed low or little correlations with walking speed, self-perceived walking impact, balance confidence, recurrent falls history (except peripheral vision), and perceived fatigue (except color vision). 

### 3.4. Multiple Linear Regression

**Figure 1:**
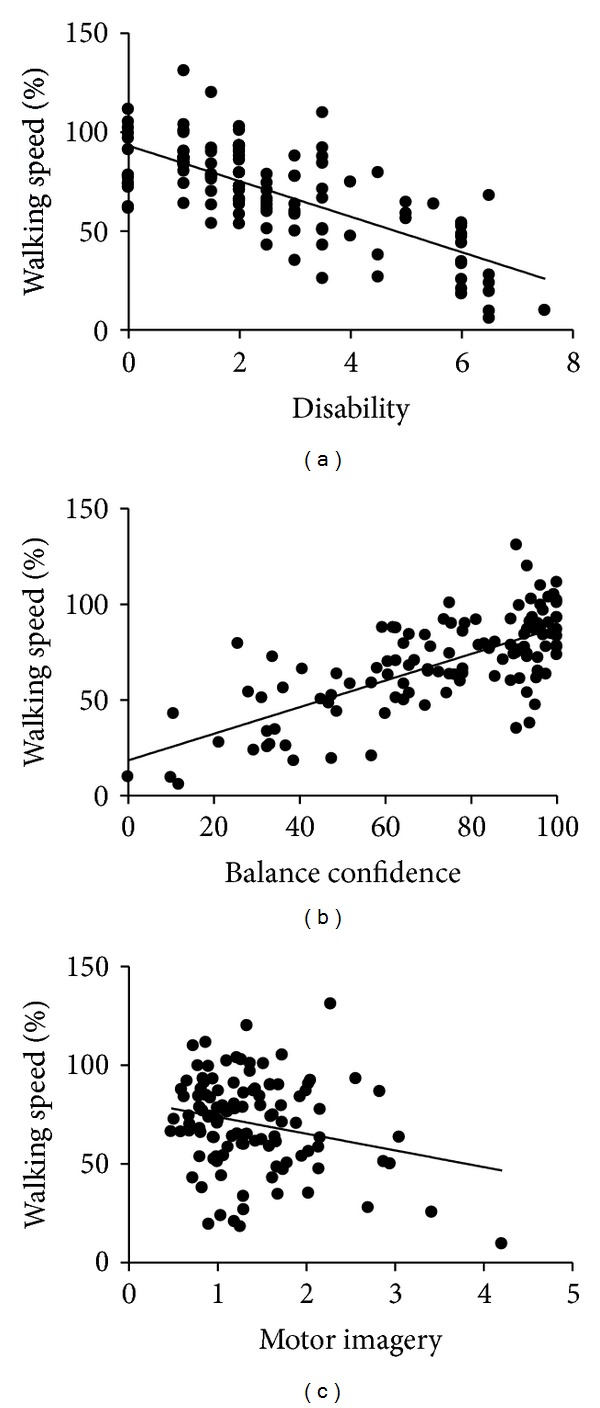
Relationship between walking speed and disability, balance confidence and motor imagery (*n* = 120 persons with multiple sclerosis).

Multiple linear regression analysis revealed that walking speed in PwMS is partially determined by disability, balance confidence, and motor planning. All together, those variables accounted for 58% of the walking speed variance (*P* = .02). After controlling for other factors, disability measured by EDSS continued to be the strongest factor correlated with walking speed (*P* < .01). Each unit increase in EDSS was associated with a 5.53% reduction in walking speed (95% CI: −7.77 to −3.29%). Furthermore, each 1% increase in balance confidence was associated with a 0.32% increase in walking speed (95% CI: 0.16 to 0.48%), as each 1% in motor planning ratio error was associated with a 3.28% reduction in walking speed (95% CI: −6.12 to −0.44%). Table 3 presents the multiple linear regression analysis results, and the scatterplots of the associations are provided in Figure 1.

Self-reported fatigue, physical activity level, age, recurrent falls history, perceived walking impact, cognitive interference, and vision-specific quality of life were not correlated with lower walking speed.

## 4. Discussion 

Walking impairment was observed in most of the participants. Fatigue, recurrent falls history, and lower balance confidence were also present. Many mobility measures were correlated with walking speed; however, disability, balance confidence, and motor planning were highly correlated with lower walking speed. It was evidenced that 85% of PwMS have lower walking speed when compared with healthy individuals within age and gender strata published in recent systematic review [36]. The lower values of walking speed may possibly reflect a more cautious walking strategy due to lack of balance confidence, cognitive, vision-specific quality of life, and fatigue impairment. 

 The demographic characteristics observed in the present study, namely, predominance of female gender, relapsing remitting evolution form, and 30–45 age group (46% of participants), are consistent with the data from the literature. Mild disability was observed in majority of the PwMS, despite the high frequency of lower walking speed. The present data found the average walking speed to be 0.95 m/s, whereas Wurdeman et al. [4] found the average walking speed to be 1.02 m/s, and even lower values with higher disability levels, corroborating our findings presented. Mild disability patients showed reduction in walking speed, when compared with healthy subjects, and higher values of disability, measured by EDSS, were found to be associated with greater mobility problems. 

Subjective fatigue is a common symptom and is negatively correlated with walking speed. Previous researches have shown moderate correlation between fatigue and walking speed in PwMS [38, 39]. Huisinga et al. [38] analyzed subjects with similar mean disability (EDSS = 2.6); however, when compared with the findings of the present study (MFIS = 32.64), their results showed a higher perceived impact of fatigue (MFIS = 42.3). Furthermore, Huisinga et al. [38] emphasized that the quality of life measures showed more relationships with gait measures than subjective fatigue rating scales, although it was hypothesized that both gait and fatigue are affected by the central neural drive. Although there might be an influence of subjective fatigue on walking speed, the mechanisms involved are not clearly understood. 

Balance impairment was also identified in the present study, as evidenced by the lack of balance confidence among PwMS and the frequent falls history. PwMS with lower walking speed presented lower balance confidence, and all fallers were classified as having lower walking speed. Matsuda et al. [14] found an association between fall status and mobility function, while Remelius et al. [2] also observed lower walking speed in PwMS with symptomatic balance problems; however, the measuring instrument used was force platform. PwMS were observed to have a greater center of pressure sway, when compared with controls [40, 41], even without balance problems in clinical tests and mild disability. Balance impairment was the strongest predictor for perceived difficulties in self-care, mobility, and domestic life [1]. Balance plays an important role in PwMS mobility and needs to be evaluated in combination with walking performance. 

Walking impact, measured by MSWS-12, was properly identified in PwMS with lower walking speed; however, interestedly, only a trend of lower level of physical activity in the same group was evident. Most of the subjects were classified to have lower walking speed and this fact can contribute to the nonsignificant difference. It has been shown that less physical activity is related to ageing, use of a cane for ambulation, unemployment, primary and secondary progressive evolution forms [42], and disability [43, 44]. To date, no study has demonstrated any relationship between physical activity level and walking speed in PwMS. The low correlation between these variables in the present study suggests that the physical activity level is not the determinant factor for walking speed in PwMS. Thus, PwMS with higher level of physical activity may not present faster walking speed than those with lower level of physical activity. Perhaps independent mobility status may play a role in walking speed, because it has been shown that independent PwMS exhibit faster walking speed, when compared with those who perceived difficulties in mobility [1]. 

Cognitive task analysis showed an interference of executive function and motor planning in walking performance. Executive function revealed a discrete influence on walking speed, whereas motor planning was overestimated significantly. Cognitive interference in walking was demonstrated in MS [45, 46], and D'Orio et al. [47] found that worse cognitive performance was related to slower walking speed. Kalron et al. [45] used a different dual-task strategy (modified word list generation test) in PwMS with a lower level of disability (mean EDSS = 1.7) and a higher walking speed (1.26 m/s) than those examined in the present study and also reported a cognitive interference in walking performance. Hamilton et al. [46] observed 12% of walking speed reduction while simultaneously performing a cognitive task, corroborating the present data (*n* = 120) that exhibited similar reduction. The present study is the first research that showed the motor planning influence on PwMS walking performance. Motor planning error assumes a central nervous system (CNS) involvement to arrange a motor task execution. Once MS directly affects the CNS, it is expected to observe a motor performance inaccuracy and higher energy expenditure, which may be related to central fatigue. 

Vision-specific quality of life was impaired in participants with lower walking speed. Little correlations were observed between vision-specific quality of life and mobility measures in the present study. To the best of our knowledge, no previous study has described the vision-specific quality of life influence on walking performance of PwMS. It has been described that a deficit of visual information had a slightly stronger impact on postural control in PwMS [40], because limiting vision increased postural instability during upright standing [41]. Healthy sighted individuals who were blindfolded showed a slower walking speed [48]. The visual influence on walking speed of healthy and other neurological conditions is well known; however, there is a lack of information on PwMS. 

Disability was the strongest factor responsible for lower walking speed, followed by motor planning and balance confidence. Previous studies described high correlations between disability and walking speed [18, 39] and found 82% of negative correlation (*r* = −0.82); on the other hand, the present study revealed 74% of negative correlation (*r* = −0.74). Our data showed that balance confidence, measured by a self-reported questionnaire, had the second highest correlation with walking speed. However, Sosnoff et al. [18] found very high correlation, using clinical tests (*r* = −0.70 and *r* = −0.90; resp.). The EDSS is a multidimensional functional system rating scale. The close relationship between walking performance and EDSS results, as described by the many relationships, supports the value of multidimensional approach to improve walking in PwMS. There has been some criticism on EDSS, and adjustments have been proposed to include potential gaps, for instance, cognitive function evaluation. A multiple linear regression model highlighted the importance of combining the multidimensional functional systems evaluation with cognitive function and balance evaluation to predict walking performance in PwMS, while no other variable contributed to this clinical outcome. Those three parameters could potentially be useful as prognostic markers of disease progression in clinical trials.

Walking speed has been widely used as an indicator of functional and physiological changes and as a predictor of falls and life expectancy [8]. Walking performance is an important issue in PwMS. The minimal detectable change value that represents whether PwMS have experienced a real change at normal walking speed has been shown to be 0.26 m/s [49]. Walking speed is easily measurable, clinically interpretable, and a potentially modifiable risk factor. Health care professionals should emphasize a multimodal approach in MS walking rehabilitation. Our results highlighted two new factors (motor planning and visual functioning) that influence PwMS walking performance, which must be investigated in future studies. One recent study described that visuoproprioceptive training increases walking speed, improves balance, and reduces risk of falls in PwMS, even in the absence of disability level changes [50].

### 4.1. Limitations

More accurate instruments should contribute to elucidate factors that affect walking speed. Physical activity level, measured by a self-report questionnaire, did not correlate with walking performance, and accelerometry may clarify the present findings. Furthermore, we suggest analyzing walking execution simultaneously with balance and motor planning evaluation, through force platform and functional magnetic resonance, respectively. Finally, muscle strength can affect walking performance in PwMS [10] and can be investigated in future studies, although this is not a unanimous result.

## 5. Conclusions

The characteristics found in patients with lower walking speed values showed multidimensional nature of walking control in PwMS. Disability, balance confidence, and motor planning were highly correlated with lower walking speed. 

## Figures and Tables

**Table 1:** Clinical characteristics of 120 persons with multiple sclerosis.

	Total (*n* = 120)	Normal walking speed (*n* = 18)	Lower walking speed (*n* = 102)	*P* value
Walking impact (MSWS-12)	28.53 ± 23.97	5.00 ± 8.26	32.20 ± 23.89	<.01*
Fallers (absolute/percentual)	36 (30.00%)	36 (35.30%)	0 (0.00%)	<.01*
Disability (EDSS)	2.68 ± 2.00	1.00 ± 1.01	3.02 ± 1.99	<.01*
Fatigue (MFIS)	32.64 ± 21.74	15.44 ± 16.67	35.67 ± 21.17	<.01*
Physical activity (IPAQ)	1991.41 ± 2567.74	3495.19 ± 3690.70	1726.04 ± 2236.41	.06
Normal walking speed (10-MWT)	0.95 ± 0.34	1.40 ± 0.14	0.86 ± 0.29	<.01*
Dual-task speed	0.84 ± 0.91	1.18 ± 0.24	0.78 ± 0.97	<.01*
Motor imagery speed (mental chronometry)	1.22 ± 0.73	1.73 ± 0.93	1.14 ± 0.66	.02*
Balance confidence (ABC scale)	72.13 ± 26.17	95.49 ± 5.95	68.01 ± 26.20	<.01*
Vision (NEI VFQ-25)				
General health	54.37 ± 29.10	77.77 ± 22.50	50.24 ± 28.25	<.01*
General vision	77.33 ± 22.44	91.11 ± 10.22	74.90 ± 23.15	<.01*
Ocular pain	79.27 ± 26.05	88.88 ± 13.48	77.57 ± 27.38	<.01*
Near activities	80.90 ± 28.03	98.61 ± 4.28	77.77 ± 29.27	<.01*
Distance activities	79.16 ± 28.08	94.90 ± 11.12	76.38 ± 29.27	<.01*
Social functioning	87.08 ± 25.56	98.61 ± 5.89	85.04 ± 27.13	<.01*
Mental health	78.02 ± 24.10	90.62 ± 8.90	75.79 ± 25.25	<.01*
Role difficulties	44.16 ± 18.40	52.08 ± 12.31	42.76 ± 18.98	.01*
Dependency	88.95 ± 25.36	99.07 ± 3.92	87.17 ± 27.09	<.01*
Color vision	92.64 ± 21.91	100.00 0.00	91.33 ± 23.56	<.01*
Peripheral vision	80.72 ± 28.75	100.00 0.00	77.25 ± 29.96	<.01*

Values expressed as mean ± SD. MSWS-12: Multiple Sclerosis Walking Scale-12. EDSS: Expanded Disability Status Scale. MFIS: Modified Fatigue Impact Scale. IPAQ: International Physical Activity Questionnaire. 10-MWT: Ten Meter Walk Test. ABC scale: Activities-Specific Balance Confidence Scale. NEI VFQ-25: Vision-specific quality of life.

*Significantly different.

**Table 2:** Mainly walking speed correlations (Pearson).

Variable	Walking speed
Disability (EDSS)	−0.740
Balance confidence (ABC scale)	0.703
Walking impact (MSWS-12)	−0.677
Motor imagery (mental chronometry)	0.556
Recurrent falls	0.445
Fatigue (MFIS)	−0.423
Physical activity (IPAQ)	0.315

All correlations are statistically significant (*P* < .01). EDSS: Expanded Disability Status Scale. ABC scale: Activities-Specific Balance Confidence Scale. MSWS-12: Multiple Sclerosis Walking Scale-12. MFIS: Modified Fatigue Impact Scale. IPAQ: International Physical Activity Questionnaire.

*Significantly different.

**Table 3:** Correlated factors with lower walking speed in persons with multiple sclerosis (*n* = 120).

Variable	Coefficient	95% Confidence interval		* P* value
		Lower	Upper
Disability (EDSS) (absolute value)	−5.53	−7.77	−3.29	<.01*
Balance confidence (ABC scale) (%)	0.32	0.16	0.48	<.01*
Motor imagery (mental chronometry) (ratio)	−3.28	−6.12	−0.44	.02*

*Statistically significant. EDSS: Expanded Disability Status Scale. ABC scale: Activities-Specific Balance Confidence Scale.
